# HRSV prefusion-F protein with Adju-Phos adjuvant induces long-lasting Th2-biased immunity in mice

**DOI:** 10.1371/journal.pone.0262231

**Published:** 2022-01-31

**Authors:** Hai Li, Hu Ren, Yangzi Zhou, Yan Zhang, Lei Cao, Wenbo Xu

**Affiliations:** 1 NHC Key Laboratory of Medical Virology and Viral Diseases, National Institute for Viral Disease Control and Prevention, Chinese Center for Disease Control and Prevention, Beijing, China; 2 Center for Biosafety Mega-Science, Chinese Academy of Sciences, Beijing, China; University of Iowa, UNITED STATES

## Abstract

The development of human respiratory syncytial virus (hRSV) vaccine has been hampered by the risk of enhanced respiratory disease (ERD) which was induced by highly skewed toward Th2 immune response. In our previous study, we expressed the recombinant pre-F protein using *Escherichia coli* BL21, called RBF. To verify if the RBF protein could cause ERD, we tested the immunogenicity and safety of RBF with a commercial alum adjuvant (GMP-grade Adju-Phos). RBF alone and RBF/Adju-Phos elicited long-lasting protective antibodies and a cellular immune response in mice after three immunizations. Unfortunately, compared with the mice in RBF group, mice in RBF/Adju-Phos generated a serious Th2 humoral immune response that elicited Th2-mediated lung pathology. From the IL-4+:IFNγ+ ratio, there was also a robust Th2 cellullar immunologic response in the RBF/Adju-Phos group. This study demonstrates that it may not be enough for RBF to increase the titer of neutralizing antibodies. A balanced immune response must be induced for hRSV vaccine safety.

## Introduction

Human respiratory syncytial virus (hRSV) is an enveloped virus belonging to the Pneumoviridae family and the Orthopneumovirus genus [1]. Sixty years ago, hRSV was identified in children admitted to a hospital in Baltimore, MD, USA, with bronchiolitis or pneumonia [2]. Since that time, hRSV has been established as a leading cause of acute lower respiratory illness (ALRI) in infants and children living in all regions of the world [3]. Nearly everyone shows evidence of an hRSV infection by the age of three [4]. However, there is still no commercially available vaccine. Prophylaxis with the humanized monoclonal antibody palivizumab is the only viable intervention for hRSV but is limited to use in high-risk infants due to its cost and modest efficacy [5]. The development of hRSV vaccines is recognized as a global priority by national governments, the World Health Organization, the pharmaceutical industry and nonprofit health organizations. Recently, approximately 60 hRSV vaccine candidates have been in development, ranging from early preclinical to pivotal phase 3 trials [6]. Among the candidates in clinical trials, nanoparticle and subunit vaccines seem to be the most promising for pregnant women and elderly patients, whereas live-attenuated or vector-based vaccines appear to be optimal for the pediatric population [7].

Many subunit vaccine candidates are currently under clinical investigation [8–10]. The most common subunit vaccine target for hRSV is the F protein, which is an envelope protein that is highly conserved across different hRSV subgroups A and B [11]. After natural infection, more than 90% of neutralizing antibodies are directed at the F protein [12]. The F protein is a trimeric glycoprotein used by the virus to enter host cells via membrane fusion and is a type I fusion protein that rearranges from a metastable prefusion conformation to a highly stable postfusion structure [13]. McLellan et al. [14] identified that prefusion F protein with epitope zero (Ø) elicited a higher titer of neutralizing activity in mice than postfusion F protein. More recently, hRSV-neutralizing antibodies, AM22, D25 and 5C4 (specific to prefusion F protein), have been found to be substantially more potent than palivizumab (which binds both pre-F and post-F proteins) [15, 16]. The target of these antibodies is antigenic site Ø (aa 62–69, aa 196–209), which is located at the apex of the prefusion glycoprotein [15]. After the discovery of the pre-F conformation, many pre-F candidates have been developed [17–19]. Uncleaved hRSV F protein retains prefusion-specific neutralizing epitopes [19] and increases antigenic stability to heat inactivation [20].

The first vaccine (formalin-inactivated RSV, FI-RSV) was evaluated in infants and young children in the 1960s. Unfortunately, this vaccine caused enhanced respiratory disease (ERD), resulting in a high rate of hospitalization and two deaths associated with peribronchiolar mononuclear cell infiltration [21]. Two features of the FI-RSV vaccine that may have contributed to ERD were the induction of antibodies with poor neutralizing activity and a Th2-polarized response characterized by cytokines associated with inflammation [22]. The Th1-biased immune response helps clear pathogens without causing inflammation or pathological damage to the lungs [23]. Based on these findings, hRSV vaccines are expected to induce antibodies with good neutralizing activity and a Th1-biased rather than a Th2-biased immune response. Adjuvants can influence the type of immune response and the titer of neutralizing antibodies and the persistence of vaccine protection. It is well known that aluminum adjuvants induce a Th2-biased immune response [24]. Monophosphoryl lipid A adjuvant with hRSV virosomes induced a Th1-skewed response [25].

The hRSV season lasts approximately 5 months each year, so clinical evaluations of many vaccines and monoclonal antibodies are also evaluated within 90 to 180 days [26, 27]. In our previous study, we found that the prefusion F proteins (RBF) expressed in *Escherichia coli* with Imject Alum adjuvant (containing aluminum hydroxide, magnesium hydroxide and inactive stabilizers [28]) induced a balanced immune response on Day 35 of immunization in mice [23]. To further verify the potential of the RBF protein as a vaccine, we verified the immunogenicity of RBF after mixing it with a GMP-grade Adju-Phos adjuvant for up to 147 days.

In this study, RBF alone and RBF/Adju-Phos elicited long-lasting protective antibodies and a cellular immune response in mice after three immunizations. However, compared with the mice in RBF group, RBF with Adju-Phos adjuvant generated robust Th2 humoral and cellullar immune responses, which elicited Th2-mediated lung pathology. RBF alone induced a balanced immune response without alveolar wall thickening, bronchiolitis or interstitial pneumonitis after hRSV infection. This study demonstrates that it may not be enough for the hRSV vaccine to increase the titer of neutralizing antibodies. A balanced immune response must be induced for hRSV vaccine safety. Therefore, a safe and effective vaccine adjuvant must be selected.

## Materials and methods

### Ethics statement

All animal experiments in this study were performed with approval from the Animal Experimental Ethical Committee of the National Institute for Viral Disease Control and Prevention (No. 20210120001). All invasive procedures were performed under anesthesia, and all efforts were made to minimize animal suffering.

### Immunization and challenge

Female BALB/C mice 6–8 weeks of age were purchased from Sibeifu Biological Technology Company (Beijing, China). We expressed the RBF proteins using *Escherichia coli* BL21 [23]. Mice were randomly distributed into experimental groups of five animals and were vaccinated on weeks 0, 3 and 6. Mice in the RBF group were vaccinated intramuscularly in the gastrocnemius of both hind legs (50 μl per leg) with 10 μg of RBF. Mice in the RBF+ Adju-Phos group were immunized with 10 μg of RBF with 100 μg of Adju-Phos adjuvant (Croda). As a negative control, one group of mice received three vaccinations with 100 μg of Adiu-Phos.

Mice were infected intranasally (i.n.) with hRSV A Long (1×10^5^ PFU) in 50 μl under anesthesia on Day 143. Bleeds were collected every 21 days using a rapid and humane method for submandibular bleeding [29]. After 10–20 min of natural coagulation at room temperature, the serum was centrifuged for 20 min (3000 rpm/min), and isolated serum was stored at -20°C until testing.

### Serum neutralization assay

hRSV-specific neutralization titers were determined by plaque reduction assays described by Garg et al. with slight modifications [30]. Briefly, 60 μL serial 4-fold dilutions of heat-inactivated (56°C for 30 min) serum were mixed with 60 μL of hRSV A Long (50 PFU) and incubated for 2 h at 37°C. The samples were then transferred to HEp-2 cell monolayers seeded in 24-well plates (Corning Costar) and incubated at 37°C for 1 h. The antibody-virus mixture was removed, and the cells were overlaid with 500 μL of 1.2% Avicel RC-591 (FMC). After 4 days, the cells were fixed and stained with 0.5% crystal violet. Neutralization titers were the dilution of serum that achieve 50% plaque reduction.

### Intracellular cytokine staining

Intracellular IL-4 and IFN-γ production by CD4+ and CD8+ T cells was evaluated using single-cell suspensions from spleens. A total of 2×10^6^ splenocytes were plated into each well and stimulated with F protein (expressed in 293F cells, 10 μg/mL) for 12 h. Then, 5 h prior to the end of stimulation, a protein transport inhibitor (1 μg/mL brefeldin A, BD Biosciences, USA) was added to each well. The cells were preincubated for 10 min with an Fc blocker (monoclonal antibody against CD16–CD32, BD Biosciences) on ice and washed with FACS buffer. The cells were labeled with mouse PE-conjugated anti-CD3 (BD Biosciences), BV421-conjugated anti-CD4 (BD Biosciences), BV510-conjugated anti-CD44 (BD Biosciences), FITC-conjugated anti-CD62 L (BD Biosciences) and PerCP-Cy5.5-conjugated anti-CD8 (BD Biosciences) surface markers for 30 min in the dark on ice. The cells were fixed with Cytofix/Cytoperm (BD Biosciences) and permeabilized with 1X permeabilization buffer (BD Biosciences). Then, the cells were incubated with APC-conjugated anti-IFN-γ and PE-conjugated anti-IL-4 (BD Biosciences) antibodies for 30 min at 4°C.

Method details for detection of virus in lung tissue, lung histopathology, serum IgG isotype antibody titers, serum IgG isotype antibody titers, statistical analysis can be found in the S1 Appendix.

## Results

### PreF/Adju-Phos elicited long-lasting antibody response

**Fig 1:**
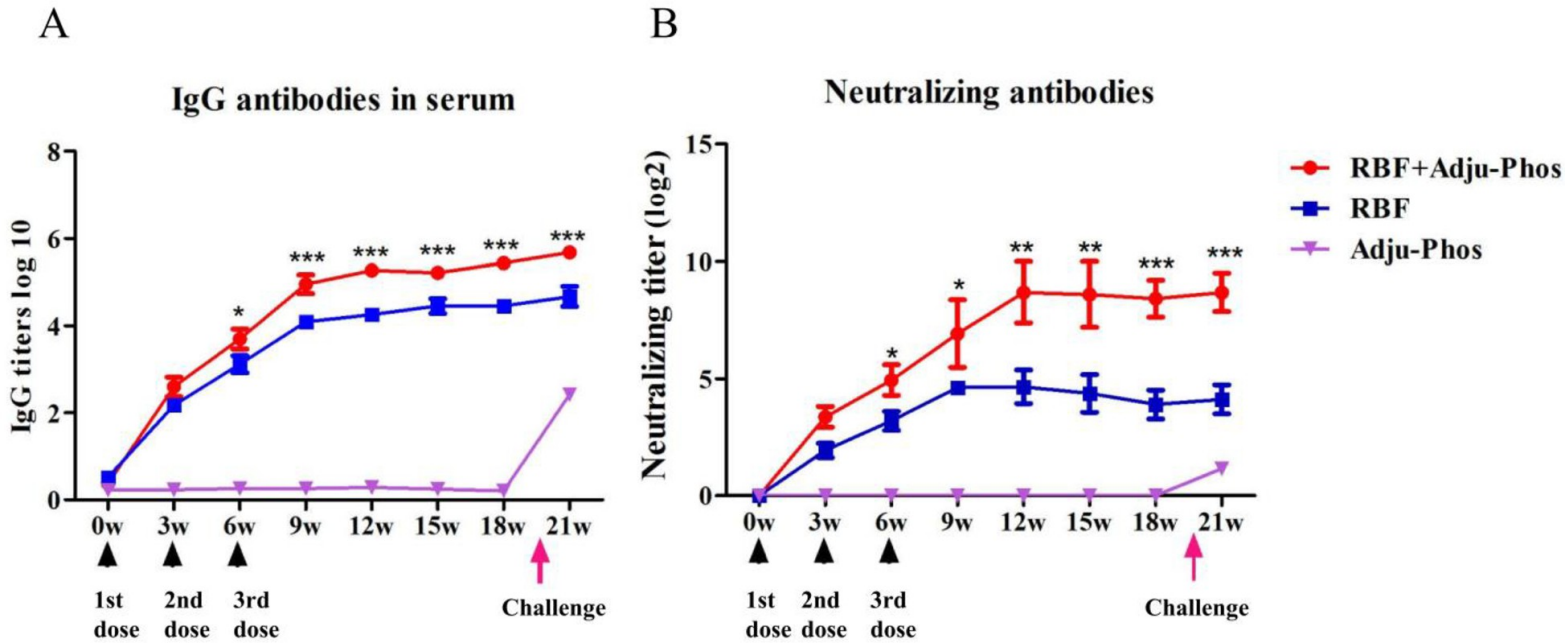
IgG-specific antibodies and neutralizing antibodies in serum. Mice were vaccinated on weeks 0, 3 and 6 and were infected intranasally (i.n.) with hRSV A Long (1×10^5^ PFU) on Day 143. Bleeds were collected every 3 weeks. (A) Dynamic changes in IgG-specific antibodies at 21 weeks. (B) Dynamic changes in neutralizing antibodies at 21 weeks. Serum neutralization titers against hRSV A Long were measured at Week 21. The titers were presented as dilution factors resulting in a 50% reduction in plaque numbers. Statistically significant differences were measured by one-way ANOVA with Newman–Keuls posttest. *** p<0.001, **p<0.01, * p<0.05.

IgG-specific antibodies were measured by indirect ELISA to determine the duration of the humoral immune response. The IgG-specific antibody titer continued to increase during the first 9 weeks of primary immunization, and the specific antibody titer in the RBF+Adju-Phos group was higher than that in the RBF group after three immunizations. On the 143rd day of the challenge, IgG-specific antibodies in the Adju-Phos immune group significantly increased after 4 days of the challenge (Fig 1A).

To appraise the quality and magnitude of the antibody response, we measured the serum-neutralizing activity of the immunized animals by plaque reduction assays. The average neutralizing antibody titer in the RBF+Adju-Phos group at week 18 was 2^9^. The titer of neutralizing antibody in the RBF group was much lower than that in the RBF+Adju-Phos group 6 weeks after primary immunization (Fig 1B).

### PreF/Adju-Phos induced the Th2-biased humoral immunity

**Fig 2:**
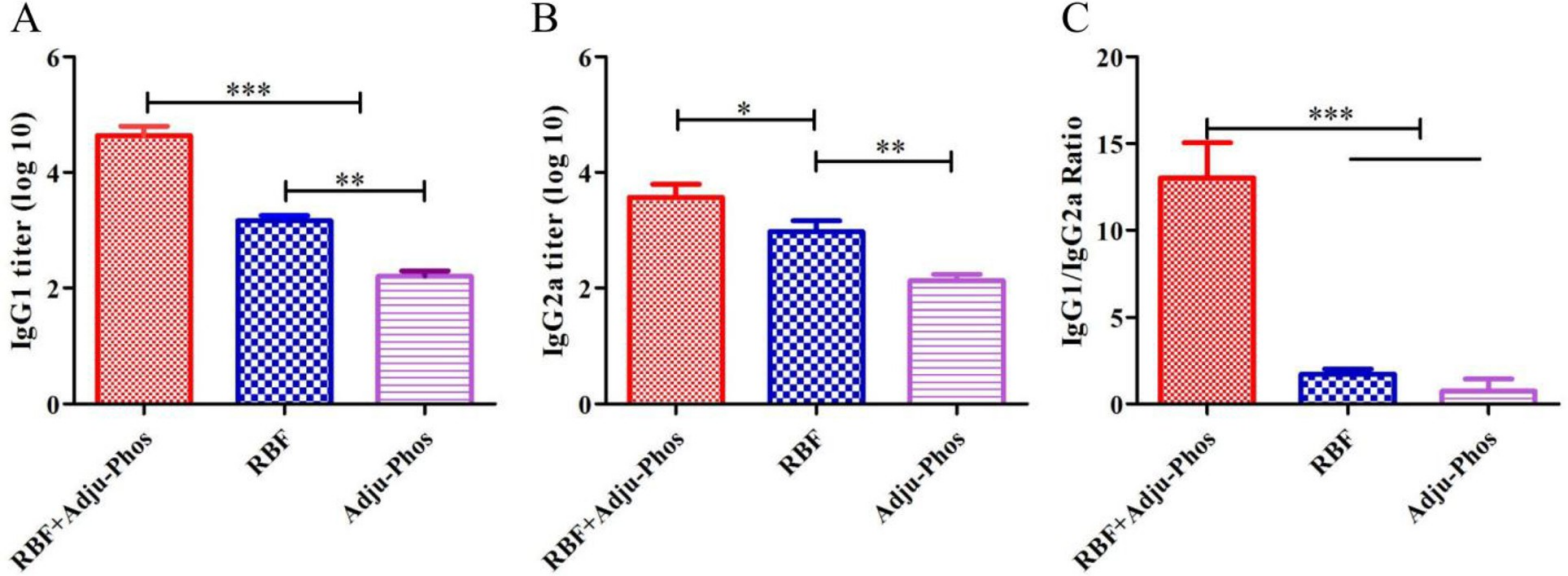
IgG1 and IgG2a titers on Day 4 after hRSV A long challenge. (A) IgG1 titers on Day 4 after hRSV A long challenge. (B) IgG2a titers on Day 4 after hRSV A Long challenge. (C) IgG1/IgG2a ratio on Day 4 after hRSV A long challenge. Statistically significant differences were measured by one-way ANOVA with Newman–Keuls posttest. *** p<0.001, **p<0.01, * p<0.05.

To evaluate the type of humoral immune response, we assessed the titers of IgG2a and IgG1 serum antibodies 4 days after hRSV challenge, which are representative of the Th1 and Th2 immune responses. The antibody titers of IgG1 and IgG2a in the RBF group were significantly higher than those in the Adju-Phos group (p<0.01) but significantly lower than those in the RBF+Adju-Phos group (Fig 2A and 2B). The IgG1/IgG2a subclass ratio in the Adju-Phos group was near 1, whereas RBF+Adju-Phos induced a higher IgG1/IgG2a ratio than RBF and Adju-Phos (Fig 2C). This result demonstrated that RBF+Adju-Phos elicited a Th2 type-dominant humoral immune response.

### PreF/Adju-Phos induced the Th2-biased cellular immunity

**Fig 3:**
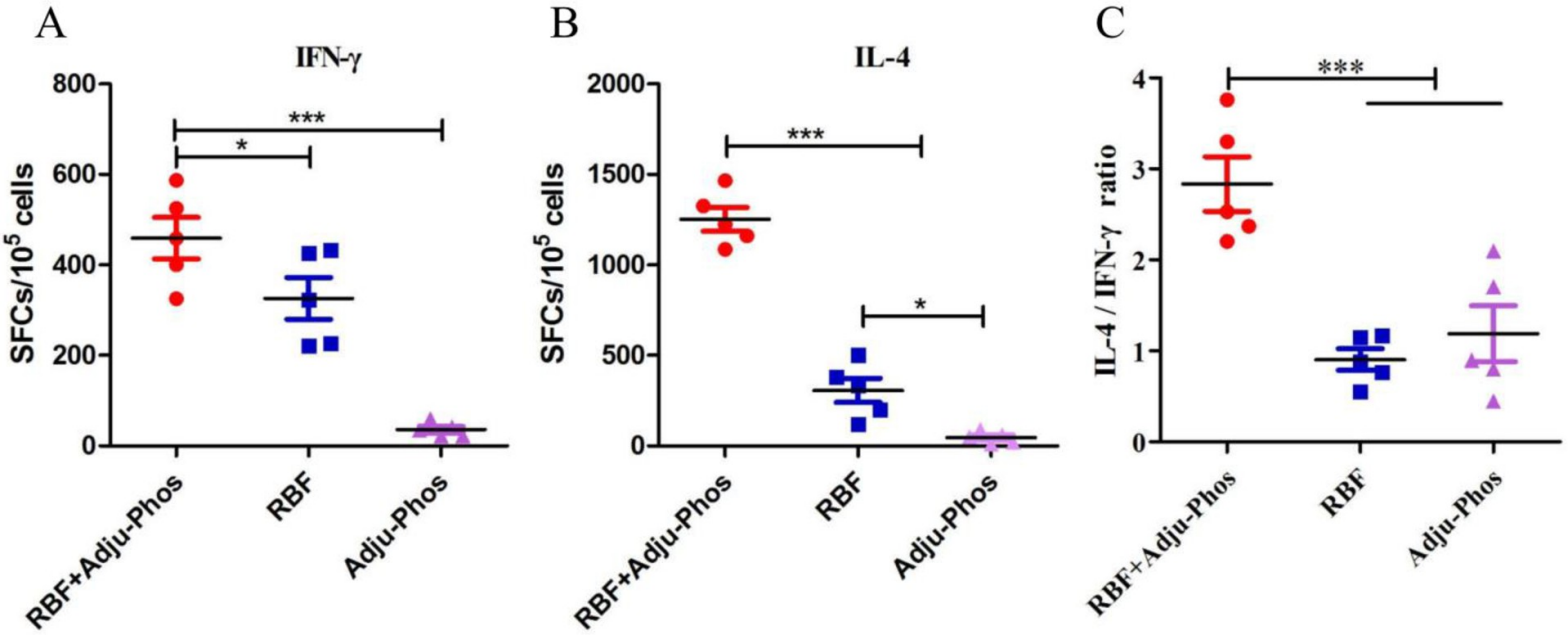
Numbers of IL-4- and IFN-γ-secreting splenocytes. ELISPOT assays were used to detect IL-4- and IFN-γ-secreting lymphocyte cells in the spleen. Splenocytes were stimulated with F protein (expressed in 293F cells, 10 μg/mL) for 16 h. (A) Numbers of IFN-γ secreting splenocytes. (B) Numbers of IL-4 secreting splenocytes. (C) Ratio of IL-4-secreting splenocytes and IFN-γ-secreting splenocytes. Statistically significant differences were measured by one-way ANOVA with Newman–Keuls posttest. *** p<0.001, **p<0.01, * p<0.05.

We investigated the number of IFN-γ- and IL-4-secreting cells in splenocytes by ELISPOT. The mice in the RBF+Adju-Phos group produced higher numbers of IFN-γ-secreting and IL-4-secreting lymphocytes than the RBF and Adju-Phos groups (Fig 3A and 3B). The IL-4-secreting lymphocyte: IFN-γ-secreting lymphocyte ratio was significantly higher than that in the RBF and Adju-Phos groups (Fig 3C). These results demonstrate that RBF induced a balanced immune response and that RBF+Adju-Phos induced a Th2-polarized immune response.

**Fig 4:**
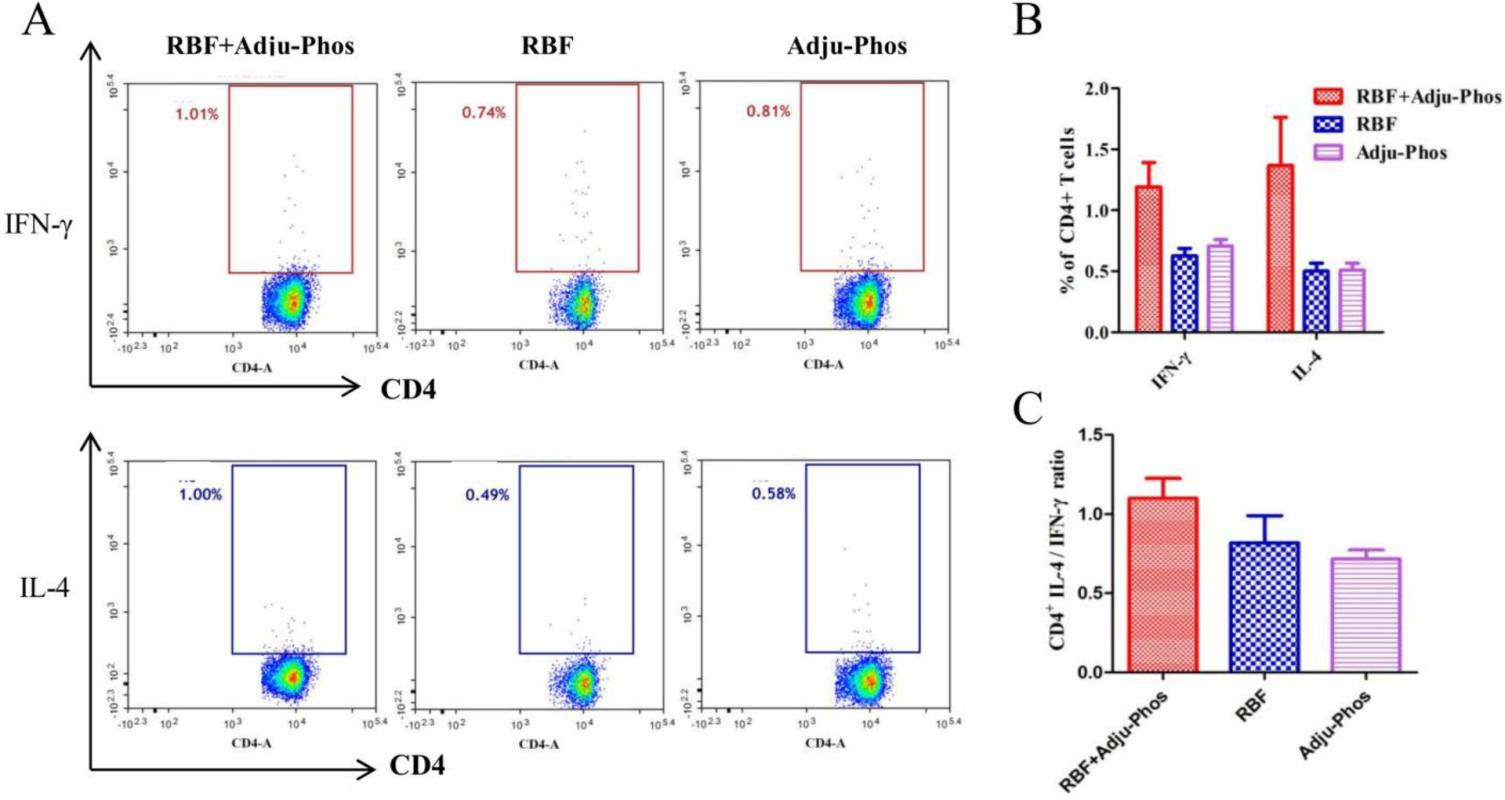
Intracellular IL-4 and IFN-γ production by CD4+ T cells. (A) The spots in the rectangular box represent IFN-γ-secreting or IL-4-secreting CD4+ T cells. (B) The percentages of CD4+ T cells producing IFN-γ or IL-4. (C) Ratio of IL-4-secreting CD4+ T cells and IFN-γ-secreting CD4+ T cells.

**Fig 5:**
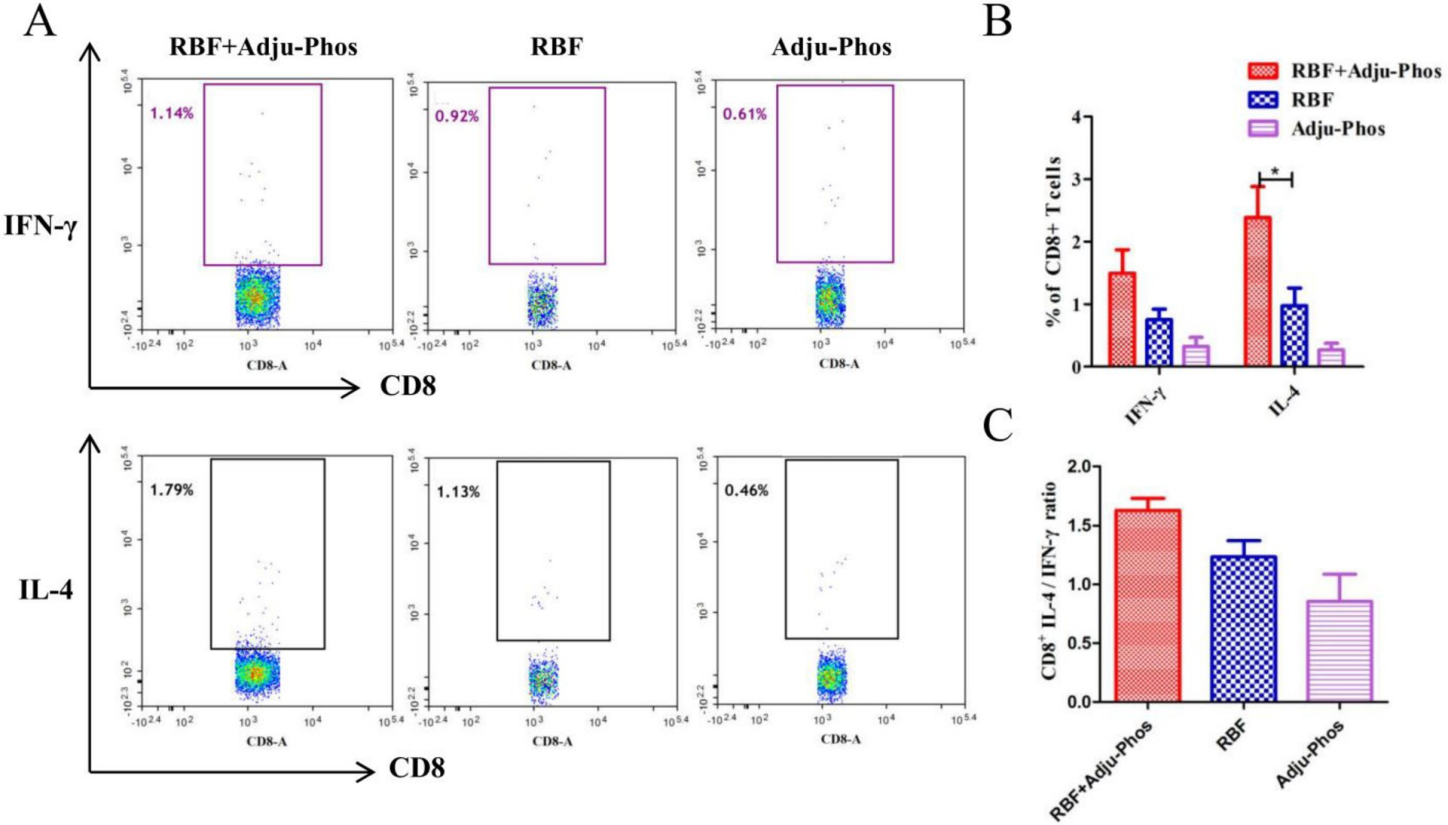
Intracellular IL-4 and IFN-γ production by CD8+ T cells. (A) The spots in the rectangular box represent IFN-γ-secreting or IL-4-secreting CD8+ T cells. (B) The percentages of CD8+ T cells producing IFN-γ or IL-4. (C) Ratio of IL-4-secreting CD8+ T cells and IFN-γ-secreting CD8+ T cells. Statistically significant differences were measured by one-way ANOVA with Newman–Keuls posttest. *** p<0.001, **p<0.01, * p<0.05.

CD4+ T cells play an important role in regulating cellular and humoral immune responses. As shown in Fig 4A and 4B, the number of IL-4-secreting and IFN-γ-secreting CD4+ T cells did not differ among the three groups. The number of IL-4-secreting CD8+ T cells in the RBF/Adju-Phos group was significantly higher than that in the Adju-Phos and RBF groups (Fig 5A and 5B). The percentage of IL-4-secreting CD8+ T cells: IFN-γ-secreting CD8+ T cells ratio in the RBF+Adju-Phos group was approximately 1.6 (Fig 5C).

### PreF/Adju-Phos elicited enhanced pulmonary inflammation

**Fig 6:**
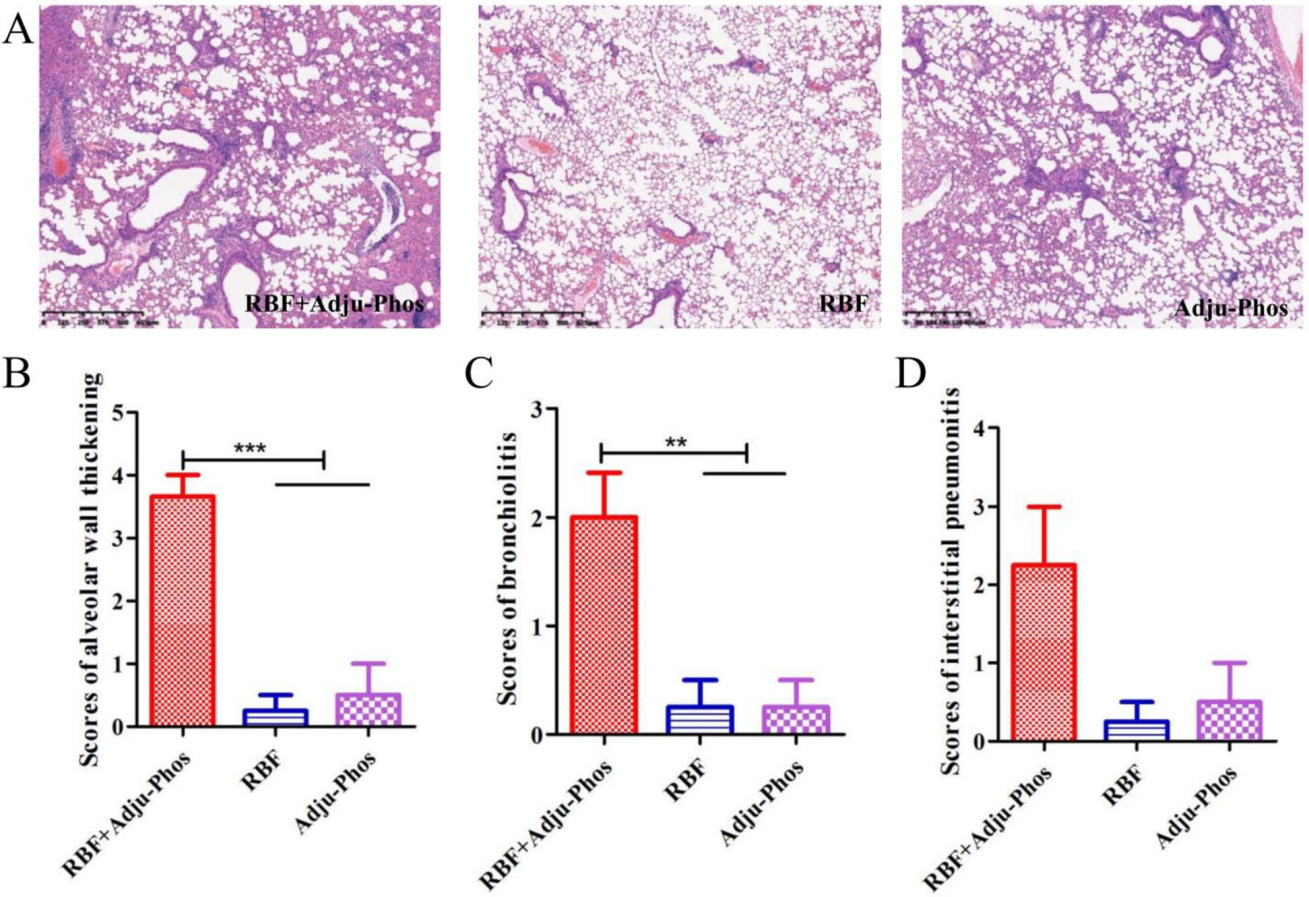
Histopathology analysis of H&E-stained. (A) Left lungs were stained with hematoxylin and eosin (H&E) for histologic evaluation. (B) Scoring of alveolar wall thickening after hRSV challenge of immunized mice. (C) Scoring of bronchiolitis after hRSV challenge of immunized mice. (D) Scoring of interstitial pneumonitis after hRSV challenge of immunized mice. Scores ranged from 0 (normal) to 4 (severe). Statistically significant differences were measured by one-way ANOVA with Newman–Keuls posttest. *** p<0.001, **p<0.01, * p<0.05.

It is expected that an hRSV vaccine with a Th1-biased response will have fewer pulmonary pathological changes after viral challenge and will not show the ERD type of changes seen in immunized mice. As shown in Fig 6A, after hRSV infection, mice immunized with RBF/Adju-Phos displayed severe bronchiolitis (Fig 6C), interstitial pneumonitis (Fig 6D) and alveolar wall thickening (Fig 6B). In contrast, we observed only slight inflammation in RBF-immunized mice and Adju-Phos-immunized mice. This result demonstrates that vaccination with the RBF proteins induces protective immunity in mice, which reduces lung injury after hRSV infection.

## Discussion

The F protein of hRSV is the major antigen used to elicit neutralizing antibody responses and protective immunity in hosts, especially for the prefusion F protein. Maternal-specific antibodies to prefusion F protein can provide immune protection for infants [31]. hRSV neutralizing antibodies can reduce the severity of the disease. In addition, the risk of reinfection was inversely associated with serum neutralizing antibody levels [32]. Therefore, improving the serum neutralizing antibody titer has always been an important goal of hRSV vaccination. Compared with the mice in RBF group, high titers of neutralizing antibodies were produced in RBF+Adju-Phos group after 3 immunizations. Most countries experience hRSV epidemics in winter, with an average duration of 10–21 weeks [33]. High titers of neutralizing antibodies and specific antibodies last for 21 weeks. Therefore, it was speculated that RBF could have a protective effect in one popular season.

CD8+ T cells play a key role in virus clearance. Specific CD8+ T cells are present in patients during primary severe hRSV infection [34]. However, CD8+ T cells have also been shown to cause immunopathology in mouse models [35]. Recent studies have shown that the presence of preneutralizing antibodies in mice prevents CD8+ T-cell-mediated immunopathology following RSV infection [36]. In this study, high titers of neutralizing antibodies were produced after immunization, so pathological damage was not caused by CD8+ T cells.

CD4+ T cells play an important role in regulating cellular and humoral immune responses. In the course of viral infection, Th2 CD4+ T cells promote the humoral immune response by secreting IL-4, IL-5, IL-10 and other Th2 cytokines. Th1 CD4+ T cells can help produce a cytotoxic CD8+ T cell response by secreting IFN-γ [37]. However, recent studies have shown that depletion of CD4+ T cells in mice completely abrogates ERD, inhibits lung inflammation and reduces immune cell infiltration. It can be assumed that CD4+ T cells promote ERD, especially Th2 CD4+ T cells [38]. In this study, the IFN-γ-secreting CD4+ T cells and IL-4-secreting CD4+ T cells in the RBF group were almost the same as those in the Adju-Phos group. However, the RBF+Adju-Phos group produced more IL-4-secreting CD4+ T cells (Fig 4). The inappropriate activation of CD4+ T cell responses may contribute to the pathology.

The FI-RSV vaccine led to ERD, which was induced by a Th2-polarized immune response and poorly neutralizing antibodies [22]. Therefore, hRSV vaccines should be designed to reduce detrimental Th2-biased immune responses and to induce a Th1-type immune response and high titers of neutralizing antibodies. After three immunizations, compared with the mice in RBF group, high titers of neutralizing antibodies were induced in the RBF/Adju-Phos group for 21 weeks. Unfortunately, based on the IgG1/IgG2a ratio, the IL-4+:IFNγ+ ratio, RBF/Adju-Phos induced predominantly Th2-biased immune responses with lung pathological damage.

Alum adjuvant can induce strong humoral immunity, which is mainly mediated by the secretion of antigen-specific antibodies, especially IgG1 [24]. Clinical trials of Novavax’s hRSV vaccine using aluminum phosphate adjuvant in pregnant women and the elderly have failed [27]. GlaxoSmithKline used aluminum adjuvant in Phase I clinical trials [39] but did not use aluminum adjuvant in Phase II clinical trials, which also showed good safety and a protective effect [40]. Similarly, in this study, RBF without Adju-Phos adjuvant induced balanced humoral and cellular immune responses without ERD.

Immunogenicity is affected not only by the concentration, dose and characteristics of the adjuvant but also by the interaction between the immunogen and the adjuvant [41]. Therefore, it is necessary to conduct adjuvant tests on subunit vaccines in preclinical studies. The ideal adjuvants for hRSV screened by previous researchers were all antigens expressed with mammalian cells [42]. The interaction between proteins expressed by different expression systems and adjuvants is different. There are some limitations in this study. First, to verify the safety and immunogenicity of the protein, we should choose the FI-RSV vaccine as the control. Second, to test the cellular immune response in mice, a total of 2×10^6^ splenocytes were stimulated with F protein for 12 h which may not be enough. Therefore, the differences in each group were not statistically significant in the flow cytometry results. In our previous study, RBF with Imject Alum adjuvant induced a balanced immune response in mice. However, RBF with Adju-Phos adjuvant generated robust Th2 immune responses and elicited Th2-mediated lung pathology. Several reasons may contribute to this result. First, the number of immunizations is different, and the Th2-biased immune response induced by three immunizations is stronger than that induced by two immunizations. Second, the age of mice is different, and usually the pathological changes of lung tissues in mice with older age are more serious [43].

Although high titers of neutralizing antibodies were induced in RBF+Adju-Phos group, a Th2-biased immune response could still lead to significant pathological damage. In the future, hRSV subunit vaccine with Th1-skewed adjuvants is a promising approach.

## Supporting information

S1 Appendix (DOC)
