# Supplementary material for: HRSV prefusion-F protein with Adju-Phos adjuvant induces long-lasting Th2-biased immunity in mice
Source: PLoS One. 2022 Jan 31;17(1):e0262231. doi: 10.1371/journal.pone.0262231 (PMC8803181; doi:10.1371/journal.pone.0262231)
Supplement: S1 Appendix — (DOC) [file pone.0262231.s001.doc]

**HRSV Prefusion-F protein with Adju-Phos adjuvant induces** **long-lasting Th2-biased immunity in mice**

**Materials and methods**

**Detection of virus in lung tissue and lung histopathology**

Four days after the hRSV challenge, the mice were anesthetized with isoflurane and exsanguinated after the caudal artery was severed. Right lungs were removed aseptically, placed in 0.5 mL of 30% sucrose in PBS, and stored at -80 ℃. After they were thawed, the lungs were weighed and then homogenized using a pestle. The homogenate was centrifuged at 2,000 rpm for 15 min, and the virus titer in the supernatant was determined by the method described above. To further verify the virus titer in the lung, cycle threshold (Ct) values were used as a semiquantitative surrogate for virus load in the lung using real-time reverse transcription polymerase chain reaction (RT–PCR). Left lungs were fixed in 10% neutral buffered formalin and stained with hematoxylin and eosin (H&E) for histologic evaluation by a board-certified pathologist on a Nikon Eclipse light microscope.

**Serum IgG isotype antibody titers**

hRSV-specific antibodies (IgG, IgG1 or IgG2a) in sera were determined by enzyme-linked immunosorbent assay (ELISA) using purified hRSV F (expressed in 293F cells, 100 ng/well) as the coated antigen. Serial 10-fold dilutions of mouse sera in PBS were added to the wells (100 μl/well) and then incubated for 1 h. HRP-conjugated goat anti-mouse IgG, IgG1 or IgG2a antibodies were used as secondary antibodies. Plates were washed twice with PBS containing 0.5% Tween 20 after each antibody incubation step. The substrate TMB was added to each well (100 μl/well) and incubated for 15–20 min at room temperature. The reaction was stopped with 2 M H2SO4, and the optical density at 450 nm was measured using an ELISA reader. Endpoint titers were calculated as the highest serum dilution that gave an optical density exceeding 2.1 times the background.

**ELISPOT assays**

ELISPOT assays were used to detect IL-4- and IFN-γ-secreting lymphocyte cells in the spleen. After dissecting the mice, the spleen was collected aseptically and fully milled 4 days after the challenge. Splenocytes were seeded at 2×10^5^ cells/well in an ELISPOT kit (Mabtech) and stimulated with F protein (expressed in 293F cells, 10 µg/mL) for 16 h in an incubator at 37 ℃ containing 5% CO_2_. Other experimental operations were performed according to the manufacturer’s instructions.

**Statistical analysis**

All data were analyzed using GraphPad Prism version 5. Differences among all groups were examined using one-way ANOVA, followed by a Newman–Keuls posttest. Differences were considered significant if P < 0.05.

**Results**


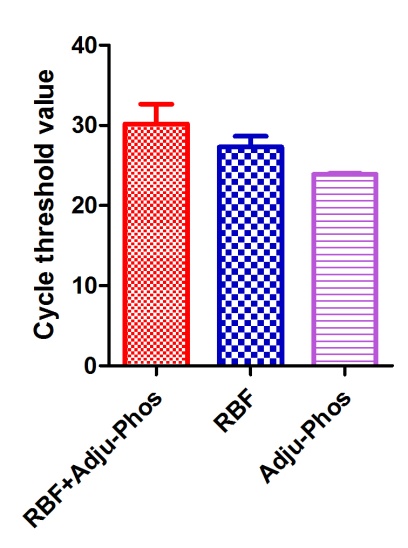


S1 Fig 1. Cycle threshold (Ct) values of real-time reverse transcription polymerase chain reaction (RT-PCR) in lung

Although an imperfect measure of virus load, Ct values were used as a semi-quantitative surrogate for virus load in lung.
